# Sleep During Oncological Treatment – A Systematic Review and Meta-Analysis of Associations With Treatment Response, Time to Progression and Survival

**DOI:** 10.3389/fnins.2022.817837

**Published:** 2022-04-19

**Authors:** Louise Strøm, Josefine T. Danielsen, Ali Amidi, Ana Lucia Cardenas Egusquiza, Lisa Maria Wu, Robert Zachariae

**Affiliations:** ^1^Unit for Psycho-Oncology and Health Psychology, Department of Psychology and Behavioral Sciences, Aarhus University, Aarhus, Denmark; ^2^Department of Psychology and Behavioral Sciences, Center for Autobiographical Memory Research, Aarhus University, Aarhus, Denmark; ^3^Aarhus Institute of Advanced Studies, Aarhus University, Aarhus, Denmark; ^4^Department of Oncology, Aarhus University Hospital, Aarhus, Denmark

**Keywords:** cancer patients, sleep, sleep-wake activity, survival, time to progression, treatment response

## Abstract

**Introduction:**

Disrupted sleep and sleep-wake activity are frequently observed in cancer patients undergoing oncological treatment. These disruptions are often associated with aggravated symptom burden and diminished health-related quality of life that in turn may compromise treatment adherence and, thus, effectiveness. In addition, disrupted sleep has been linked to carcinogenic processes, which ultimately could result in worse prognostic outcomes.

**Aims:**

Our aim was to systematically review and conduct a meta-analysis of studies examining the associations between sleep and sleep-wake activity and prognostic outcomes in cancer patients undergoing oncological treatment.

**Methods:**

A comprehensive systematic search of English language papers was undertaken in June 2020 using PubMed, The Cochrane Library, and CINAHL. Two reviewers independently screened 4,879 abstracts. A total of 26 papers were included in the narrative review. Thirteen papers reporting hazard ratios reflecting associations between a dichotomized predictor variable (sleep) and prognostic outcomes were subjected to meta-analysis.

**Results:**

Nineteen of the 26 eligible studies on a total of 7,092 cancer patients reported associations between poorer sleep and poorer response to treatment, shorter time to progression, and/or reduced overall survival, but were highly heterogeneous with respect to the sleep and outcome parameters investigated. Meta-analysis revealed statistically significant associations between poor self-reported sleep and reduced overall survival (HR = 1.33 [95% CI 1.09–1.62], *k* = 11), and shorter time to progression (HR = 1.40 [95% CI 1.23–1.59], *k* = 3) and between poor objectively assessed sleep and reduced overall survival (HR = 1.74 [95% CI 1.05–2.88], *k* = 4).

**Conclusion:**

The current findings indicate that disturbed sleep during treatment may be a relevant behavioral marker of poor cancer prognosis. The limited number of studies, the common use of single item sleep measures, and potential publication bias highlight the need for further high quality and longitudinal studies.

## Introduction

Disturbances and alterations in sleep architecture and behavior commonly occur when individuals experience medical illness (Opp and Krueger, 2015), and cancer is no exception (Clevenger et al., 2012; Sharma et al., 2012; Loh et al., 2018). Such sleep disturbances are often associated with aggravation of symptom burden (Palesh et al., 2010; George et al., 2016) and impairments to quality of life (Trudel-Fitzgerald et al., 2014; Nho et al., 2017). Additionally, accumulating evidence highlights the important role of healthy sleep in cell genome stability (Lamia, 2017), efficient immune responses (Fondell et al., 2011; De Lorenzo et al., 2015), and sufficient melatonin secretion that can mitigate carcinogenic processes (Schernhammer and Schulmeister, 2004; Mirza-Aghazadeh-Attari et al., 2020). Hence, sleep disturbances and disorders have been linked to various pathologies, including increased risk of cancer (Erren et al., 2016; Mogavero et al., 2021), all-cause mortality in the general population (Dew et al., 2003), and tumor progression in mouse models (Papagiannakopoulos et al., 2016; De Lorenzo et al., 2018). Moreover, once diagnosed with cancer, the cancer itself may serve as an indirect factor influencing sleep, through various pathophysiological processes, including inflammation, which has been proposed as an underlying biological mechanism of sleep disturbance (Raison et al., 2010; Irwin et al., 2016; Besedovsky et al., 2019). This suggests that the cancer-related inflammatory response may be an additional contributor to alterations in cancer patients’ sleep (Mantovani et al., 2008). Apart from biological mechanisms, psychological symptoms, such as heightened stress, depression, and anxiety may also play an important role in the manifestation of sleep disturbance (Ancoli-Israel, 2009; Liu et al., 2009). In addition, persistent behavioral problems, including those arising from pediatric cancers, may contribute to long-term sleep disturbance (Mogavero et al., 2020). However, both psychologically and biologically driven sleep disturbances are most likely bi-directionally related, both contributing to the perpetuation and exacerbation of sleep and sleep-wake irregularity (Krueger et al., 2003, 2009; Meier-Ewert et al., 2004; Rockstrom et al., 2018; Ashok Kumar et al., 2019), which makes the relationship between sleep, sleep-wake activity, tumorigenesis and cancer progression highly complex.

While sleep disturbances may be present in cancer patients already prior to treatment (Zhou et al., 2018), many patients undergoing oncological treatment, especially chemotherapy and radiotherapy, experience sleep problems with prevalence estimates ranging from 30% to 75% (Ancoli-Israel et al., 2001; Savard et al., 2009, 2015; Palesh et al., 2010; Costa et al., 2014). Cancer prognosis has improved for most cancers, especially with the introduction of new targeted therapies like immune checkpoint inhibitors (Kennedy and Salama, 2020). However, sleep disturbances during treatment in these patients could challenge response to treatment and compromise survival, by potentially aggravating symptom burden, hence compromising adherence to treatment (Kidwell et al., 2014), as well as potentially disrupting immunological and endocrine processes in protecting the body against cancer development (Eismann et al., 2010). In a meta-analysis by Stone et al. (2019), long sleep duration was found to be associated with increased cancer-specific mortality for all-cancers, and all-cause mortality for breast cancer. While this review added to the field by highlighting the long-term risks of sleep duration on cancer-specific mortality, it focused on sleep duration in cancer survivors both pre-diagnosis and years after treatment completion. Other meta-analyses investigating sleep and cancer-mortality have been limited, primarily focusing on general population samples (Gallicchio and Kalesan, 2009; Ma et al., 2016). Thus, to the best of our knowledge, no review has been published on the association between sleep disturbance during oncological treatment and the subsequent response to treatment, time to progression and survival. However, this is an important time period in the trajectory of cancer patients, since an increased symptom burden including sleep disturbance may have prognostic consequences.

Therefore, the primary aim of the present review was to systematically review and conduct a meta-analysis of available studies examining associations between disturbed sleep and sleep-wake activity and cancer prognostics, e.g., treatment response, time to progression, and survival in a population of cancer patients undergoing oncological treatment.

Improving our knowledge about the association between sleep during treatment and treatment response and survival, could enable the development of targeted interventions to support patients’ recovery, at a critical time in the course of their disease.

## Methods

### Registration and Search Strategy

This present systematic review and meta-analysis adheres to the Preferred Reporting Items for Systematic Reviews and Meta-Analyses (PRISMA) guidelines (Moher et al., 2009), and was pre-registered in PROSPERO (Page et al., 2018) (registration ID: CRD42020189880). A broad systematic search of English language papers was undertaken by the first author and a librarian in June 2020 using PubMed, The Cochrane Library, and CINAHL. The following search terms were used, including MeSH-terms or MeSH-term equivalents: (Sleep OR insomnia OR “circadian rhythm” OR rhythm) AND (immunotherapy OR immunotherapies OR “checkpoint inhibitor” OR “checkpoint inhibitors” OR ICI OR ICIs OR “PD-1” OR “PD-L1” OR nivolumab OR pembrolizumab OR chemotherapeutic OR chemotherapy OR “cancer treatment*” OR cytostatic) AND (effect OR effects OR outcome OR outcomes OR “clinical response” OR “clinical effect” OR response OR “response to treatment” OR survival OR mortality OR prognos*) AND (cancer OR neoplasms OR neoplasm). No publication date restriction was imposed.

### Selection Criteria and Screening

Identified records were imported to the review software Covidence (Kellermeyer et al., 2018). Two authors (LS and JTD) independently screened 4,879 abstracts, according to predefined hierarchically displayed inclusion criteria based on the PICO framework (Sackett et al., 1996) and adapted to meet our research question: “Is sleep and sleep-wake activity in cancer patients receiving oncological treatment associated with response to treatment, time to progression and survival?”. Inclusion criteria were: (1) cancer patients regardless of diagnosis, who have received approved oncological treatment, except for transplantation; (2) all sleep-related measures (self-reported and objective) represented by at least one quantified item, assessed after diagnosis, and immediately prior to or during cancer treatment; (3) outcome evaluated in relation to a sleep measure (4) outcome constitutes clinical response to oncological treatment, overall survival or time-to-progression after oncological treatment; and (5) all types of observational and controlled trial studies of adult humans (≥18 years), except for Phase 1 studies.

If abstracts reported both a sleep measure and measure of treatment response or survival indicating that results on a possible association could be found in the full text, studies were considered eligible for full text review. Full texts were independently screened by two authors (LS and JTD), and reasons for exclusion were documented. Conflicts were resolved at consensus meetings with a third author (RZ). Subsequently, reference lists of included studies and relevant reviews were screened for papers missed by the systematic database search. Moreover, included studies were objects for citation searches.

### Quality Assessments

Methodological quality and risk of bias assessment, was undertaken independently by two authors (LS and JTD) for all included studies, using the NIH Quality Assessment Tool for Observational Cohort and Cross-Sectional Studies (National Heart Lung, 2014). Although 12 studies based their results on samples obtained in randomized controlled trails, the measurement of sleep data and outcome met criteria for observational designs.

### Data Extraction and Synthesis

Data extraction was performed independently by two authors (LS and AC). The data extraction form included: name of first author, year of publication, title of paper, study design, number of participants, age and gender of participants, diagnosis of participants, treatment regimens, treatment-naivety, method used for assessing sleep or sleep-wake activity (i.e., subjective/objective assessment), time of reported sleep measure (i.e., prior to treatment, during treatment), sleep measure (actigraphy measure, sleep scale or sleep item), number of assessments, outcome measure (i.e., response rate, response classification, time to progression/progression free survival, and overall survival) results, and median follow-up time. In case of missing data, the corresponding author of set paper was contacted for this information. A narrative method was applied to synthesize the findings, and an *a priori* decision was made to perform a meta-analysis if a minimum of three studies reported comparable predictor and outcome measures. Analyses were grouped according to the predictor being self-reported or objective and according to the reported treatment outcome (i.e., overall survival, time-to-progression and response to treatment).

### Meta-Analytic Strategy

A total of 13 studies were subjected to meta-analysis to ascertain the pooled overall effect estimate and its precision. Eligibility criteria were results of unadjusted analyses reported as hazard ratios reflecting associations between a dichotomized predictor variable (sleep) and the outcome. One study reporting only adjusted analyses (Sullivan et al., 2006), was also included. To aid the interpretation of the results, we conducted a Bayesian Model-Averaged meta-analysis, as a supplement to the conventional frequentist meta-analysis (Gronau et al., 2017). The frequentist analyses were performed using Comprehensive Meta-Analysis, version 3 (Borenstein et al., 2013). The supplementary Bayesian analyses were conducted with JASP Version 0.12.2 (JASP Team, 2022).

#### Pooling Effects

An inverse variance-weighted random-effects model considering the precision of each study was used in all analyses, with hazard ratios larger than 1.0 taken to indicate an effect in the hypothesized direction, i.e., poor sleep associated with a shorter time to progression or shorter overall survival. Three studies reported survival outcomes for several sleep variables (Palesh et al., 2014; Cash et al., 2018; Gottfried et al., 2020). For these studies, the results were combined into one pooled weighted result to ensure independence of effects included in the meta-analysis.

#### Heterogeneity

Heterogeneity was investigated using Q and *I*^2^ statistics (Higgins et al., 2019). Heterogeneity tests aim at determining to which degree the variation in effect sizes reflects true differences (heterogeneity) or sampling error. The *I*^2^ value is an estimate of the between-study variance in a pooled effect estimate that is accounted for by heterogeneity of the effect sizes in the included studies and is assumed to be relatively unaffected by the number of studies (Higgins et al., 2003). If the results indicated heterogeneity (*I*^2^ > 0.0), we calculated the 95% prediction interval, which estimates the expected range of true effects in 95% of similar future studies (IntHout et al., 2016).

#### Publication Bias

The possibility of publication bias was assessed using funnel plots and Egger’s test (Egger et al., 1997). If results were suggestive of possible publication bias, sensitivity analyses were conducted by imputing the “missing studies” and calculating adjusted effect estimates using the Duval and Tweedie trim-and-fill method (Duval and Tweedie, 2000).

#### Moderator Analysis

To explore possible sources of heterogeneity (*I*^2^ > 0.0), we examined the role on the effect size of four possible moderators with meta-regression based on random-effects models and estimated with maximum likelihood method. The moderators included percent women, median follow-up time in months, mean sample age, and cancer stage (advanced vs. mixed). If associations were found between the moderators, this was adjusted for in the analysis.

#### Bayesian Analysis

A supplementary Bayesian Model-Averaged meta-analysis (Gronau et al., 2017) of the associations between sleep and overall survival and time-to-progression, respectively, examined the results of four models: (a) fixed-effect null hypothesis (fH_0_), (b) fixed-effect alternative hypothesis (fH_1_), (c) random-effects null hypothesis (rH_0_), and (d) random effects alternative hypothesis (rH_1_). Bayesian Model-Averaged analysis thus avoids selecting either a fixed- or random-effects model and addresses two questions in light of the observed data: What is the plausibility that the overall effect is non-zero and is there between-study variability in the effect size? We chose an uninformed prior probability, i.e., 25%, of each of the four models and 2,000 iterations. Concerning parameter distributions, we chose previously recommended defaults (Gronau et al., 2017). We thus used a zero-centered Cauchy prior with a scale of 0.707 for the effect size. To have zero indicating the null effect, the hazard ratios and the upper and lower limits were log-transformed. For the between-study variation, we used an empirically informed prior distribution of non-zero between-study deviation estimates based on effect sizes from 705 meta-analyses published in Psychological Bulletin between 1990 and 2013 (van Erp et al., 2017). This distribution has been approximated by an Inverse-Gamma (1, 0.15) prior on the standard deviation (Tau) (Gronau et al., 2017).

## Results

### Search Results

**FIGURE 1:**
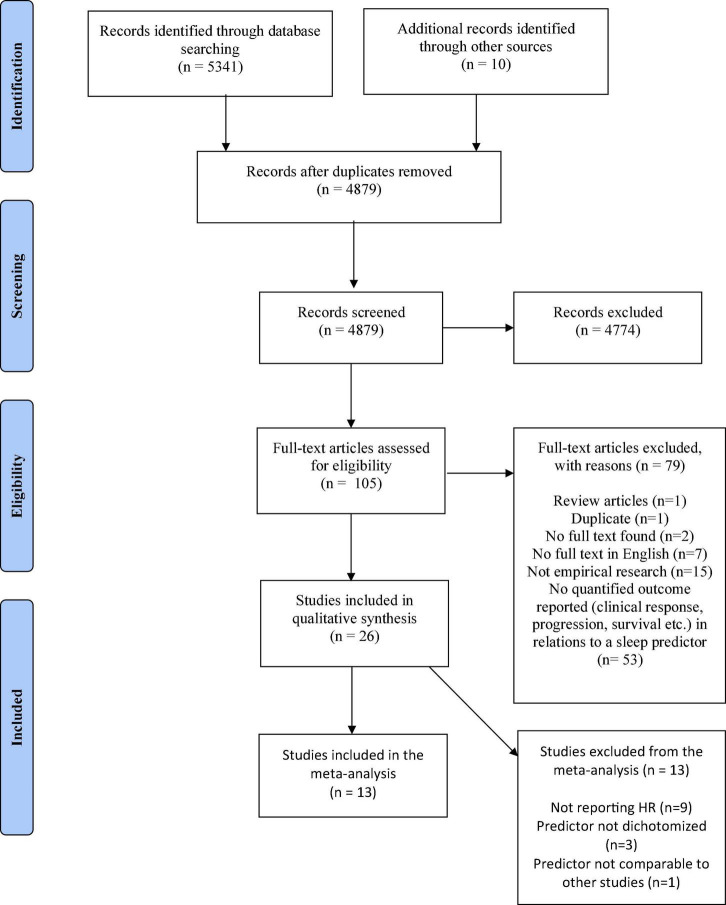
PRISMA flow diagram.

A total of 4,879 studies were identified after duplicate removal and 10 additional studies were identified by other sources (two studies through reference list screenings, and eight studies by citation search) of which five were eligible for full text screening. A total of 105 papers were eligible for full text screening with 26 studies being included for analysis. Exclusion of the 79 studies upon full-text screening were primarily attributed to the exclusion criteria “not relating a sleep measure to one of the predefined outcome measures, e.g., response to treatment, time to progression or overall survival” (67%). A table of all excluded studies following full-text screening including reasons for exclusion are provided in the Supplementary Table 1. Level of conflict following the abstract screening was 103 out of 4,879 screenings corresponding to a 97.9% agreement between reviewers. Following full text, agreement was 93.3% (Cohen’s Kappa 0.82). Full-text conflicts were mainly concerned with which primary exclusion criterion to apply. Screening and selection process is provided in the PRISMA flow diagram in Figure 1.

### Study Characteristics

Characteristics of the 26 included studies are shown in Table 1 and described in the sections below.

**TABLE 1:** Study characteristics.

Study	Cancer type	Study design	N in analysis (% women)	Sleep (predictor)	Timing of sleep assessment	Prognostic outcomes	Median follow-up (months)	Analysis and predictor	Results (Direction of association^1^)	Included in meta-analysis (+)
				(S) = Self-report						
				(O) = Objective						
Braun et al., 2011	Non-small cell	Observational –	1194 (49.7%)	EORTC QLQ-C30 (S)	Pre-treatment	Overall survival	NR	**Unadjusted:** Insomnia	ns	
	lung cancer	retrospective						**Adjusted:** NR		
								**Unadjusted:**		
Braun et al., 2012	Prostate	Observational –	673 (0%)	EORTC QLQ-C30 (S)	Pre-treatment	Overall survival	NR	Insomnia	ns	
	cancer	retrospective						**Adjusted:** Insomnia	ns	
								**Unadjusted:**		
Cash et al., 2018	Head and	Observational –	38 (40%)	Actigraphy (O)	Pre-treatment	Overall survival	24	Sleep-wake activity (r24)	**+**	+
	neck cancer	prospective						Sleep-wake activity (I < 0)	**+**	
						Response to treatment		Sleep-wake activity (I < 0)	**+**	
								**Adjusted:**		
						Overall survival		Sleep-wake activity (I < 0)	**+**	
								**Unadjusted:**		
Chandra et al., 2019	Hematological	Observational –	66 (40.90%)	PSQI (S)	Pre-treatment	Overall survival	6	Sleep quality	**+**	
	malignancy and lymphoma	prospective						**Adjusted:** NR		
								**Unadjusted:**		
Chang and Lin, 2014	Lung, breast,	Observational –	68 (50%)	Actigraphy (O)	During treatment	Overall survival	84	Sleep-wake activity (I < 0)	**+**	+
	gastro and	retrospective		PSQI (S)				Sleep quality	**+**	
	liver, head and							**Adjusted:**		
	neck cancer,							Sleep-wake activity (I < 0)	**+**	
	hematology, genitourinary							Sleep quality	ns	
								**Unadjusted:**		
Collette et al., 2004	Prostate	RCT	388 (0%)	EORTC QLQ-C30 (S)	Pre-treatment	Overall survival	NR	Insomnia	**+**	+
	cancer							**Adjusted:**		
								Insomnia	**+**	
								**Unadjusted:**		
Collins et al., 2017	Hepatobiliary-	Observational –	292 (36%)	PSQI (S)	NR	Overall survival	NR	Sleep latency	ns	
	pancreatic	retrospective						Sleep efficiency	ns	
	system							Shorter sleep duration	**+**	
	cancers as							Shorter and longer sleep duration	**+**	
	primary							**Adjusted:**		
	cancers or							Shorter sleep duration	**+**	
	metastases from other primary cancers							Shorter and longer sleep duration	**+**	
								**Unadjusted:**		
Geels et al., 2000	Breast cancer	RCT	198 (NR)	QoL and CRF (case report forms) (S)	Pre- and during treatment	Response to treatment	NR	Insomnia (QoL) Insomnia (CRF)	ns ns	
								**Adjusted:** NR		
								**Unadjusted:**		
Gottfried et al., 2020	Lung cancer	Registry-based	404 (40.80%)	Single sleep question (S)	Pre-treatment	Overall survival	26	Difficulty falling asleep	ns	+
								Frequent arousals at night	**+**	
								Both of the above combined	**+**	
								**Adjusted models:**		
								Sleep abnormalities	**+**	
								**Unadjusted:**		
Innominato et al., 2009	Colorectal	RCT	130 (43.10%)	Actigraphy (O)	Pre- and during	Overall survival	72	Sleep-wake activity (I < 0)	**+**	
	cancer				treatment			Sleep-wake activity (r24)	**+**	
								Mean rest-activity rhythm	ns	
						Response to treatment		Sleep-wake activity (I < 0 and r24)	ns	
						Progression free survival		Sleep-wake activity (I < 0 and r24)	ns	
								**Adjusted models:**		
						Overall survival		Sleep-wake activity (I < 0)	**+**	
								**Unadjusted:**		
Innominato et al., 2012	Colorectal cancer	RCT	77 (35.10%)	Actigraphy (O)	During treatment	Overall survival	77.2	Sleep-wake activity (I < 0)	+	+
						Response to treatment & Progression free survival		Sleep-wake activity (I < 0)	ns	
								**Adjusted:**		
						Overall survival		Sleep-wake activity (I < 0)	+	
								**Unadjusted:**		
Innominato et al., 2015	Colorectal	RCT	361 (38.80%)	EORTC	Pre- and during	Overall survival	89.2	Insomnia	**+**	+
	cancer			QLQ-C30 (S)	treatment	Time to progression		Insomnia	**+**	
						Response to treatment		Insomnia	**+**	
								**Adjusted:**		
						Overall survival		Insomnia	**+**	
								**Unadjusted:**		
Kramer et al., 2000	Breast cancer	Observational –	187 (100%)	EORTC QLQ-C30 (S)	Pre-treatment	Overall survival	42.2	Insomnia	ns	+
		retrospective				Response to treatment		Insomnia	ns	
								**Adjusted:** NR		
								**Unadjusted:**		
Kuo et al., 2020	Lung cancer	Observational –	33 (18.20%)	Actigraphy (O)	Pre-treatment	Overall survival	6.15	Sleep-wake activity (I < 0)	**+**	+
		prospective						**Adjusted:**		
								Sleep-wake activity (I < 0)	**+**	
								**Unadjusted:**		
Lévi et al., 2014	Colorectal	RCT	436 (37.40%)	Actigraphy (O)	Pre-treatment	Overall survival	NR	Sleep-wake activity (I < 0)	**+**	
(Cohort III)^2^	cancer					Progression-free survival (PFS)		Sleep-wake activity (I < 0)	**+**	
								**Adjusted:**		
						Overall survival		Sleep-wake activity (I < 0)	**+**	
								**Unadjusted:**		
Maisey et al., 2002	Colorectal	RCT	501 (37%)	EORTC QLQ-C30 (S)	Pre-treatment	Overall survival	34.1	Insomnia	**+**	+
	cancer							**Adjusted:**		
								Insomnia	**+**	
								**Unadjusted:**		
Merli et al., 2004	Non-Hodgkin’s lymphoma	RCT	91 (66%)	EORTC QLQ-C30 (S)	Pre-, during and post-treatment	Treatment response	NR	Insomnia	**+**	
								**Adjusted:** NR		
								**Unadjusted:**		
Mormont et al., 2000	Colorectal	Observational –	192 (33%)	Actigraphy (O)	Pre-treatment	Overall survival	24	Sleep-wake activity (I < 0)	**+**	
	cancer	prospective				Objective response		Sleep-wake activity (I < 0)	**+**	
								Sleep-wake activity (r24)	**+**	
								**Adjusted:**		
						Overall survival		Sleep-wake activity (I < 0)	**+**	
								Sleep-wake activity (r24)	**+**	
								Mean sleep-wake activity	**+**	
								**Unadjusted:**		
Naughton et al., 2002	Small-cell lung	RCT –	67 (29%)	Sleep Quality Scale –	Pre- and during	Overall survival	NR	Sleep quality	ns	
	cancer	companion study		four items (S)	treatment			**Adjusted:** NR		
								**Unadjusted:**		
Nowak et al., 2004	Pleural	Phase II trial	53 (15%)	EORTC QLQ-C30 (S)	Pre-treatment	Overall Survival	NR	Insomnia	ns	
	mesothelioma	single-arm						**Adjusted:** NR		
								**Unadjusted:**		
Palesh et al., 2014	Breast cancer	Observational –	97 (100%)	Sleep logs and	NR	Overall survival	72	Sleep efficiency	**+**	+
		prospective		actigraphy (S), (O)				Time in bed	ns	
								WASO	**+**	
								Wake episodes	**+**	
								Wake episode duration	**+**	
								Sleep latency	ns	
								**Adjusted:**		
								Sleep efficiency	+	
								WASO	+	
								Wake episodes	+	
								Wake episodes duration	+	
								**Unadjusted:**		
Robinson et al., 2012	Endometrial	Cross-	453 (100%)	EORTC QLQ-C30 (S)	During and after	Overall survival	NR	Insomnia (Ovarian cancer)	ns	+
	and ovarian	sectional			treatment			Insomnia (Endometrial cancer)	ns	
	cancer							**Adjusted:**		
								Insomnia (Ovarian cancer)	ns	
								Insomnia (Endometrial cancer)	ns	
								**Unadjusted:**		
Roychowdhury et al., 2003	Bladder	RCT	363 (20.80%)	EORTC QLQ-C30 (S)	Pre-treatment	Overall survival	NR	Insomnia	**+**	+
	cancer					Time to progression		Insomnia	**+**	
						Time to treatment failure		Insomnia	ns	
								**Adjusted:** NR		
								**Unadjusted:**		
Sullivan et al., 2006	Prostate	RCT	765 (0%)	EORTC QLQ-C30 (S)	Pre- and during		NR	NR		+
	cancer				treatment			**Adjusted:**		
						Overall survival		Insomnia	ns	
								Change in insomnia	**+**	
						Time to progression		Change in insomnia	**+**	
								**Unadjusted:**		
Teunissen et al., 2004	Gastro-entero-	Observational –	42 (56%)	EORTC QLQ-C30 (S)	Pre- and post-treatment	Treatment response	1.2	Change in insomnia	**+**	
	pancreatic cancers	prospective						**Adjusted:** NR		
								**Unadjusted:**		
Zhao et al., 2013	Breast, lung, lymphoma, alimentary tract or nasopharynx cancer	Observational – Open label trial	240 (47%)	PSQI (S)	Pre- and post-treatment	Treatment response	NR	Sleep quality Change in sleep quality **Adjusted:** NR	**+** **+**	

*^1^Please note that + = Poor sleep → worse prognosis. − = Poor sleep → improved prognosis; ns, non-significant association. ^2^Reported on the independent sample in the study, to avoid sample overlap with other studies. EORTC-QLQ-30, European Organization for the Research and Treatment of Cancer Core Quality of Life Questionnaire; NR, not reported; PSQI, The Pittsburgh Sleep Quality Index; RCT, randomized controlled trial; WASO, wake after sleep onset; Sleep-wake activity (r24), autocorrelation coefficient at 24 h (regularity and reproducibility of activity pattern); Sleep-wake activity (I < 0), dichotomy index (relative ratio of activity in and out of bed).*

#### Diagnosis and Stage

A total of 13 different cancer diagnoses were represented in the 26 studies. The most frequently investigated cancers were colorectal cancer reported in six studies (Mormont et al., 2000; Maisey et al., 2002; Innominato et al., 2009, 2012, 2015; Lévi et al., 2014), non-small cell lung cancer in six studies (Naughton et al., 2002; Braun et al., 2011; Zhao et al., 2013; Chang and Lin, 2014; Gottfried et al., 2020; Kuo et al., 2020), and breast cancer in five studies (Geels et al., 2000; Kramer et al., 2000; Zhao et al., 2013; Chang and Lin, 2014; Palesh et al., 2014). In four studies, the sample consisted of mixed cancer populations (Zhao et al., 2013; Chang and Lin, 2014; Collins et al., 2017; Chandra et al., 2019). Twenty-four studies included patients with advanced disease, of which 10 studies included mixed cancer stages, and two studies failed to report cancer stage (Geels et al., 2000; Chandra et al., 2019).

#### Study Samples

The sample sizes ranged from 33 to 1,194 (median = 190). Three studies reported data from the same trial, but had different study objectives and no overlapping data (Innominato et al., 2009, 2012, 2015). Thus, they were considered independent samples. One paper (Lévi et al., 2014) reported a pooled sample, consisting of three different samples. Two of these samples were already represented in our review (Mormont et al., 2000; Innominato et al., 2009), thus, only the third and newly obtained sample from the paper was included. One study (Robinson et al., 2012) reported separate data for two independent samples of women with ovarian and endometrial cancer.

#### Study Design and Treatment Regimen

Twelve of the studies reported their sample to be subsamples from Phase III randomized controlled trials and were therefore combined samples in which different treatment regimens were utilized. Treatment regimens were reported in 20 studies, of which 18 reported having different regimens within the same study, including systemic treatment (i.e., hormonal and chemotherapy), radiation or surgery. Seven of the 26 study samples included treatment-naive participants at study entry. Thus, in the majority of studies, participants had received oncological treatment prior to participation.

#### Quality Assessments

Supplementary Table 2 provides an overview of the quality ratings for the individual studies. Overall, the ratings indicated high quality regarding clear definitions of research question, study population, exposure and outcome measures, and high quality in assessing exposure prior to outcome, as well as including a sufficient timeframe between the two. However, assessment of sleep more than once had low-quality ratings in all but five studies, and although all studies meet criteria for examining effects of different levels of sleep disturbance on outcome, levels were converted to a dichotomous variable in 16 studies, whereas six studies used quartiles, composite scores or change in symptom score between two assessments. Continuous variables were used in four studies. Moreover, power justifications were only reported in two studies.

#### Sleep Parameters

Both self-reported (*k* = 19) and objective (*k* = 7) measures were used to evaluate sleep, and one study (Palesh et al., 2014) reported a self-reported measure verified by an objective measure. Of the 18 studies only using self-reported sleep measures, the majority (*k* = 13) were based on a single item regarding sleep disturbances from the European Organization for the Research and Treatment of Cancer Core Quality of Life Questionnaire (EORTC QLQ-C30), a validated quality of life instrument for cancer patients (Aaronson et al., 1993). In this item cancer patients respond to the following question about their sleep: “During the past week, have you had trouble sleeping?”. The four available response categories are; “Not at all,” “a little,” “quite a bit,” and “very much” (Aaronson et al., 1993). The Pittsburgh Sleep Quality Index (PSQI), a 19-item self-rated questionnaire that assesses sleep quality and disturbances over a 1-month period was used in three studies (Zhao et al., 2013; Collins et al., 2017; Chandra et al., 2019). One study (Gottfried et al., 2020) reported sleep based on one of two questions: “difficulty falling asleep?” and “frequent arousals at night?”. The last study (Naughton et al., 2002) assessed sleep using a single item about “trouble sleeping”, but this was not clearly described.

In all of the studies with objective evaluations of sleep (*k* = 7), the sleep outcome was sleep-wake activity measured with actigraphy [i.e., a small non-invasive wrist-worn activity logger (Ancoli-Israel et al., 2003)]. Sleep-wake activity was evaluated using the dichotomy index I < 0, measuring the relative amount of activity in bed versus out of bed (Ortiz-Tudela et al., 2014). Three studies included an additional evaluation—the autocorrelation coefficient r24— measuring the regularity of the activity pattern over 24 h (Ortiz-Tudela et al., 2014).

#### Associations Between Sleep, Treatment Response, Time to Progression and Survival

Reported prognostic outcomes included treatment response, time to progression or overall survival. Survival was the most frequent outcome (*k* = 22), and nine of the 26 studies reported on more than one outcome. Overall, 19 of 26 studies found poor sleep to be significantly associated with poorer survival or worse treatment response, while seven studies found no associations. Poor sleep was assessed from both self-reported (e.g., insomnia symptoms, poor sleep quality) and actigraphy-derived sleep outcomes (e.g., sleep-wake activity, nighttime restfulness, wake after sleep onset, sleep latency, sleep efficiency, and wake episodes). No studies found associations between better sleep and poorer treatment response, shorter time to progression or poorer survival. Effect sizes and sample sizes differed significantly with hazard ratios ranging from 1.10 to 13.70, and sample sizes ranging from 33 to 1,194. Only two studies examined sleep duration. In one study of patients with advanced cancers affecting the hepatobiliary and pancreatic systems, both short and long sleep duration were associated with increased mortality (linear term: hazard ratio = 0.485; quadratic term: hazard ratio = 1.064) (Collins et al., 2017), but in another study of women with advanced breast cancer, there was no linear or quadratic association between sleep duration and survival (Palesh et al., 2014).

Adjusted models were reported in 16 of the 26 papers. However, covariates were neither comparable in characteristic nor in number, and only selected models were subjected to adjustments. In all but one study (Chang and Lin, 2014) results of the adjusted models remained statistically significant.

### Meta-Analytic Results

#### Association Between Self-Reported Sleep and Overall Survival

**FIGURE 2:**
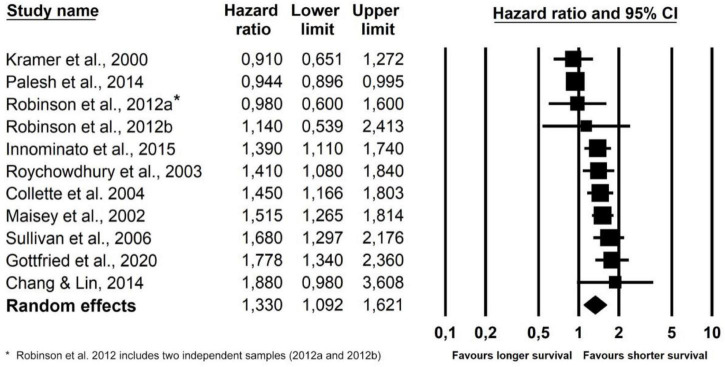
Forest plot of poorer self-reported sleep and overall survival.

**FIGURE 3:**
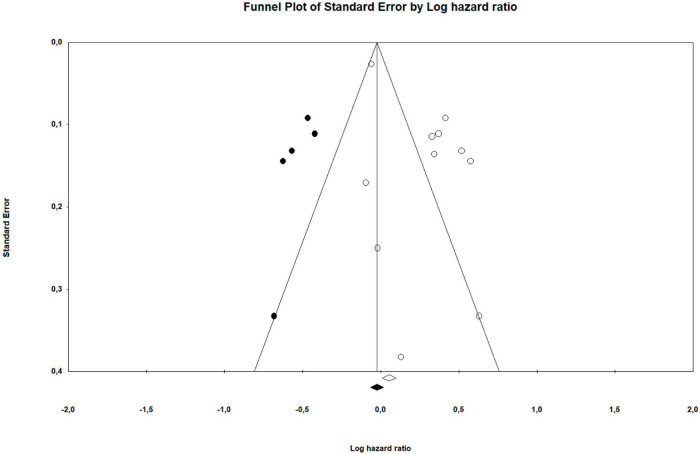
Funnel plot of suggested publication bias.

As seen in Table 2 and Figure 2, the overall combined hazard ratio for the 11 studies investigating self-reported sleep and overall survival in a total of 3,050 patients was 1.33, indicating that poorer self-reported sleep was statistically significantly associated with reduced overall survival (95% CI: 1.09–1.62; *p* = 0.005). A sensitivity analysis omitting Sullivan et al. (2006), which had only used an adjusted model, resulted in a similar pooled effect size (HR = 1.30; 95% CI: 1.06–1.59; *p* = 0.012). A visual inspection of the funnel plot and the statistically significant Egger’s test (*p* = 0.012) suggested the possibility of publication bias in the direction of an association in the hypothesized direction. As seen in Figure 3, the trim and fill method yielded five “missing” studies, which, when imputing these values, resulted in a smaller effect reduced to statistical non-significance. The high *I*^2^ (87.6%) suggests that a considerable proportion of the variance is explained by systematic differences between studies rather than sampling error. When exploring the influence of possible between-study differences on the association between poorer self-reported sleep and reduced overall survival with meta-regression, the associations were significantly weaker in studies with a larger percentage of women (Slope: −0.006: *p* < 0.001). As seen in Table 3, the association was maintained when adjusting for cancer stage. While cancer stage was not a significant moderator of the association when adjusting for the percentage of women in the studies, a statistically weaker association (slope: −0.24; *p* = 0.032) was found between poorer sleep and reduced overall survival in studies of patients with advanced cancer, compared to studies with mixed samples. The remaining moderating factors explored did not reach statistical significance. The findings of the frequentist analysis were supported by the supplementary Bayesian Model-Averaged meta-analysis, which provided strong evidence for a non-zero effect of poor self-reported sleep on overall survival corresponding to a Bayes Factor (BF) (Duval and Tweedie, 2000) of 10.2, i.e., indicating that the alternative hypothesis is 10.2 times more likely than the null-hypothesis. The Bayesian analysis provided very strong evidence concerning heterogeneity of the effects. The BF for heterogeneity was 10^12^ indicating that the probability that the effect sizes are heterogeneous are extremely likely.

**TABLE 2:** Meta-analysis of associations between poorer self-reported sleep and overall survival and time to progression, and between poorer objective sleep and overall survival.

				Heterogeneity^c^				Pooled results			
Predictor	Outcome	K^a^	N^b^	Q	*p*	*I* ^2^	Tau^2^	HR^d^	95%CI	*p*	95%PI^e^
Self-reported sleep	Overall survival	11	3050	80.7	<0.001	87.6	0.083	1.33	1.09–1.62	**0.005**	0.67–2.65
	*Adj. for publication bias* ^f^	*(16)*	–	–	–	–	–	*1.02*	*0.85–1.23*	*NS*	–
	Overall survival (sensitivity analysis)^g^	8	2,787	11.4	0.121	38.8	0.012	1.37	1.21–1.56	**<0.001**	1.01–1.87
	Time to progression	3	1,489	0.53	0.766	0.0	0.0	1.40	1.23–1.59	**<0.001**	–
Objective sleep	Overall survival	4	216	13.4	0.004	77.6	0.176	1.74	1.05–2.88	**0.032**	0.20–14.45
	Overall survival (sensitivity analysis)^h^	3	183	9.3	0.010	78.5	0.131	1.54	0.96–2.46	0.071	–

*^a^K = number of studies in the analysis; One study reported data for two independent samples (Robinson et al., 2012). ^b^N = total number of participants in the analysis. ^c^Q-statistic: p-values < 0.10 taken to suggest heterogeneity; I^2^ indicates the proportion of the variance in effect sizes explained systematic (non-random) between-study differences. ^d^HR = hazard ratio with a value > 1 indicating an association between worse sleep and negative prognosis. ^e^PI = 95% prediction interval: The interval in which 95% of future observations will fall, given the observed data, calculated for heterogeneous ESs (I^2^ > 0). ^f^In case of statistically significant ESs and possible publication bias (Egger’s test statistically significant (p < 0.05), “missing studies” are imputed and an adjusted pooled ES calculated (Duval and Tweedie, 2000). ^g^Sensitivity analysis, including only studies based on the EORTC QLQ-C30 questionnaire. ^h^Sensitivity analysis omitting Kuo et al. (2020), an outlier with a HR (5.57) approaching (93%) two standard deviations from the pooled HR.*

**TABLE 3:** Exploring moderators of the association between poorer self-reported sleep and overall survival.

Moderator	K*^a^*	Slope*^b^*	95%CI	*p*
Percent women	11	−0.006	−0.007 to −0.005	**<0.001**
Percent women (adjusting for stage)	11	−0.006	−0.007 to −0.004	**<0.001**
Follow-up (months)	6	−0.003	−0.011 to 0.006	0.545
Sample mean age	5	0.032	−0.011 to 0.076	0.142
Advanced stage vs. mixed (ref.)	11	−0.117	−0.467 to 0.234	0.514
Advanced stage vs. mixed (ref.) (adjusting for percent women)	11	−0.24	−0.474 to 0.021	**0.032**

*^a^Analyses conducted when K ≥ 5. ^b^Mixed effects regression (unrestricted ML).*

#### Associations Between Self-Reported Sleep and Time to Progression

**FIGURE 4:**
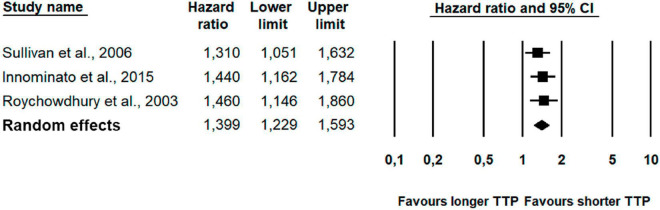
Forest plot of poorer self-reported sleep and time to progression (TTP).

As seen in Table 2 and Figure 4, the overall combined hazard ratio for the three studies investigating self-reported sleep and time to disease progression in a total of 1,489 patients showed that poor self-reported sleep was statistically significantly associated with shorter time to progression (HR: 1.40; 95% CI: 1.23–1.59; *p* < 0.001). The findings of the frequentist analysis were supported by the supplementary Bayesian Model-Averaged meta-analysis, which provided strong evidence for a non-zero effect of poor self-reported sleep on time to progression corresponding to a BF (Duval and Tweedie, 2000) of 20.9, i.e., indicating that the alternative hypothesis is more than 20 times more likely than the null-hypothesis. The BF for non-heterogeneity was 2.5, providing only anecdotal evidence for non-heterogeneous effect sizes. Due to the small number of studies, publication bias and moderator analyses were not conducted.

#### Associations Between Objectively Assessed Sleep and Overall Survival

**FIGURE 5:**
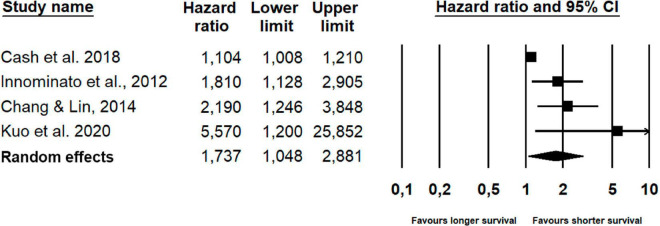
Forest plot of poorer objective sleep and survival.

As seen in Table 2 and Figure 5, the overall combined hazard ratio for the four studies investigating objectively assessed sleep and overall survival in a total of 216 patients showed that poor objective sleep was statistically significantly associated with reduced overall survival (HR: 1.74; 95% CI: 1.05–2.88; *p* = 0.032). However, a sensitivity analysis omitting an outlier with a HR (5.57) approaching two standard deviations from the pooled HR, resulted in a smaller, and non-significant, effect. The supplementary Bayesian meta-analysis provided only anecdotal evidence for a non-zero effect of poor objectively assessed sleep on overall survival corresponding to a BF (Duval and Tweedie, 2000) of 2.3, i.e., indicating that the alternative hypothesis is only 2.3 times more likely than the null-hypothesis. The Bayesian analysis provided strong evidence for heterogeneity of the effects with a BF of 10.3. Due to the small number of studies, publication bias and moderator analyses were not conducted.

## Discussion

### Summary of Main Findings

To the best of our knowledge, this is the first systematic review of literature on associations between sleep immediately prior to or during treatment in cancer patients and prognostic indicators, thus filling a knowledge gap on the role of sleep during a critical period in the cancer trajectory. Overall, the findings of the narrative part of this review suggests that disturbances in sleep and sleep-wake activity immediately prior to or during treatment are associated with reduced overall survival, poorer response to treatment, and shorter time to progression. Traditional frequentist meta-analyses with Bayesian meta-analysis, provided evidence in support of poorer self-reported sleep being associated with reduced overall survival and shorter time to progression. However, these findings should be interpreted with caution due to the indications of possible publication bias with respect to the analyses of self-reported sleep, and less robust results with respect to the association between objective sleep and overall survival. Moderator-analyses showed a significantly weaker association between poor self-reported sleep and reduced overall survival in studies with a higher percentage of women. Furthermore, when adjusting for the percentage of women in the studies, a weaker association between poor self-reported sleep and reduced survival was found in samples of patients with more advanced cancer. One explanation for the moderating effect of female sex in the context of breast cancer could be that while anti-hormonal treatments improve prognosis, they at the same time induce menopausal symptoms that may interfere with sleep, e.g., hot flashes (Desai et al., 2013), thus weakening the association between sleep disturbance and poor prognosis. No studies reporting associations between sleep and response to treatment were eligible for meta-analysis, due to heterogeneity in reported sleep parameter, outcome, and analytic strategy.

### Strengths, Limitations, and Future Perspectives

Several strengths of this review should be noted. First, previous reviews have primarily focused on the association between sleep duration and cancer-mortality in the general population (Gallicchio and Kalesan, 2009; Ma et al., 2016) and in cancer survivors (Stone et al., 2019). The present review of the role of disrupted sleep and sleep-wake activity, thus, provides a more nuanced picture of sleep assessed during a critical period in the cancer trajectory and its associations with prognostic outcomes. Second, the present review and meta-analysis included both self-reported and objective sleep parameters, providing a broader scope on sleep. Although related and to some extent inter-dependent (Choilek et al., 2021), self-reported and objective parameters represent qualitatively different aspects of sleep (Acker et al., 2021). Actigraphy is typically used to objectively examine sleep-wake-activity and sometimes external light conditions and temperature as proxies for determining sleep and wake periods (Acker et al., 2021), whereas self-reported evaluations of sleep capture the experienced sleep and sleep disturbance and related effects/side-effects (Bastien et al., 2001; Choilek et al., 2021), and have been shown to be influenced by mood to a larger degree than objective evaluations (Baillet et al., 2016). One type of measure is not necessarily better than the other, but merely highlights the multi-modality of the sleep construct. Both aspects should be taken into account to obtain the most accurate picture of a persons’ sleep. Third, our comprehensive search strategy highlighted the fact that the majority of studies (*k* = 20) were not primarily designed to examine sleep or sleep disturbances in these patients. Our review thereby provides evidence of the need for rigorous longitudinal studies focusing on sleep and sleep-wake activity across the course of treatment and the relationship with prognostic outcomes.

A number of limitations highlighted by the present review should be mentioned. First, although a variety of measures of sleep disturbance were examined in this manuscript, which provided a more nuanced picture of sleep during the cancer trajectory (including self-reported and actigraphy-based measurements of sleep disturbances), their diversity also weakens comparability across studies. Second, a majority of the reviewed studies (19 out of 26) were based on self-reported sleep outcomes, with more than half of these relying on only a single item from the EORTC QLQ-C30. While self-report measures of sleep using multi-item scales such as the Insomnia Severity Index and Pittsburgh Sleep Quality Index have been shown to have good psychometric properties (Beck et al., 2004; Morin et al., 2011), it is unclear whether single-item assessments such as the sleep item from the EORTC QLQ-C30 exhibits the same level of sensitivity and reliability in detecting sleep disturbance, which could compromise the validity of the results. Nevertheless, a sensitivity analysis that only included studies using the single sleep item from the EORTC QLQ-C30, revealed a similar hazard ratio. Third, of the more objective sleep measures included, none consisted of polysomnography, which could have provided important details about underlying sleep architecture. Fourth, we could not include studies of sleep duration in the meta-analysis highlighting the need for further work in that area (Palesh et al., 2014; Collins et al., 2017). Fifth, we were not able to conduct a meta-analysis on the association between sleep and response to treatment due to heterogeneity in study methodology. However, it is worth noting that recent lines of evidence suggest that treatment response may be modulated by therapies affecting sleep and circadian rhythms. For example, administration of melatonin, as an adjuvant cancer therapy, may improve the effectiveness and reduce the side-effects of radio- and chemotherapies through several mechanisms, including stimulation of apoptosis and inhibition on angiogenesis (Li et al., 2017; Farhood et al., 2019; Mortezaee et al., 2019). Moreover, chronotherapies that take advantage of the control of the circadian system may modulate the pharmacokinetic properties of antitumoral agents, thus optimizing their efficacy and reduce toxicity (Ozturk et al., 2017). Sixth, only overall survival was reported in the available studies. Hence, whether these studies reflect associations between sleep and cancer-specific mortality is not clear. Seventh, only half of the studies were eligible for meta-analysis, due to heterogeneity in the analytic strategies, sleep measures and outcome parameters used, thereby limiting our interpretability of our findings. Finally, 80% of studies reported associations based on a single time-point measurement collected immediately prior to or during treatment. However, sleep disturbances have been found to fluctuate both during and after treatment, and may, for some groups, improve over time (Thomas et al., 2010; Savard et al., 2011). Moreover, when compared to inconsistent sleep patterns, more regular sleep behavior has been shown to be associated with lower risk of cancer-specific mortality (Marinac et al., 2017). Thus, single-time point sleep measures may be limited in their ability to predict survival, treatment response and time to progression. This also limits our ability to infer any causal relationship, leaving the question of causality unanswered: Do sleep disturbances reflect disease and symptom burden, or are sleep disturbances disrupting otherwise protective biological mechanisms and compromising treatment efficacy? Our results thus highlight the importance of continuing to investigate the effect of sleep on prognostic outcomes. Finally, future research should also examine sleep in a broader range of cancer populations, as cancer treatment varies according to cancer type.

## Conclusion

In sum, this review and meta-analysis points to disturbances in sleep and sleep-wake activity as potential predictive markers of reduced survival, poorer response to treatment and shorter time to progression in cancer patients undergoing oncological treatment, though findings ought to be interpreted with caution due to issues with heterogeneity and methodology. Prospective longitudinal studies investigating fluctuations in sleep across the course of treatment and its relationship with prognostics are warranted.

## Data Availability Statement

The original contributions presented in the study are included in the article/Supplementary Material, further inquiries can be directed to the corresponding author.

## Author Contributions

All authors contributed to the protocol of this systematic review. The literature search and data exportation were performed by LS and librarian Gina Bay, and titles and abstracts were screened by LS and JTD. LS and JTD performed full text review and quality assessments, validated by RZ, LW and AA. Data extraction was performed by LS and ALCG, and RZ and LS were responsible for the analyses. LS and RZ wrote the manuscript, and all authors critically revised the manuscript and approved the final version.

## Conflict of Interest

The authors declare that the research was conducted in the absence of any commercial or financial relationships that could be construed as a potential conflict of interest.

## Publisher’s Note

All claims expressed in this article are solely those of the authors and do not necessarily represent those of their affiliated organizations, or those of the publisher, the editors and the reviewers. Any product that may be evaluated in this article, or claim that may be made by its manufacturer, is not guaranteed or endorsed by the publisher.
