# Supplementary material for: Sleep During Oncological Treatment – A Systematic Review and Meta-Analysis of Associations With Treatment Response, Time to Progression and Survival
Source: Front Neurosci. 2022 Apr 19;16:817837. doi: 10.3389/fnins.2022.817837 (PMC9063131; doi:10.3389/fnins.2022.817837)
Supplement: Supplementary file 1 [file Table_1.docx]

**Table S1.** Excluded studies after full-text screening including reasons for exclusion.

| **Article** | **Reasons for exclusion** | |
| --- | --- | --- |
| #5319 - Atzpodien 2003  Atzpodien, J; Küchler, Th; Wandert, T; Reitz, M  Rapid deterioration in quality of life during interleukin-2- and alpha-interferon-based home therapy of renal cell carcinoma is associated with a good outcome  British journal of cancer 07// 2003;89(1):50-54  Nature Publishing Group 2003 07/ | 13. No quantified outcome measure reported (clinical response / prognosis / Progression free survival / survival etc.) in relation to a sleep measure | |
| #844 - Awan 2017  Awan, FT; Thangavadivel, S; Weiss, D; Wei, L; Woyach, JA; Rogers, KA; Jones, J; Andritsos, L; Muthusamy, N; Andersen, B; et al.  A phase 2 trial of early intervention with ibrutinib in patients with asymptomatic, high-Risk CLL  2017; 130():  201 | 4. Not primary research (empirical research study | |
| #4927 - Bacon 2003  Bacon, M; James, K; Zee, B  A comparison of the incidence, duration, and degree of the neurologic toxicities of cisplatin-paclitaxel (PT) and cisplatin-cyclophosphamide (PC).  International journal of gynecological cancer : official journal of the International Gynecological Cancer Society // 2003;13(4):428-434  2003 // | 13. No quantified outcome measure reported (clinical response / prognosis / Progression free survival / survival etc.) in relation to a sleep measure | |
| #2585 - Burke 2018  Burke, John M; Shustov, Andrei; Essell, James; Patel-Donnelly, Dipti; Yang, Jay; Chen, Robert; Ye, Wei; Shi, Wen; Assouline, Sarit; Sharman, Jeff  An Open-label, Phase II Trial of Entospletinib (GS-9973), a Selective Spleen Tyrosine Kinase Inhibitor, in Diffuse Large B-cell Lymphoma.  Clinical lymphoma, myeloma & leukemia 08// 2018;18(8):e327-e331  2018 08// | 13. No quantified outcome measure reported (clinical response / prognosis / Progression free survival / survival etc.) in relation to a sleep measure | |
| #3969 - Castillo 2018  Castillo, Jorge J; Meid, Kirsten; Gustine, Joshua N; Dubeau, Toni; Severns, Patricia; Hunter, Zachary R; Yang, Guang; Xu, Lian; Treon, Steven P  Prospective Clinical Trial of Ixazomib, Dexamethasone, and Rituximab as Primary Therapy in Waldenström Macroglobulinemia.  Clinical cancer research : an official journal of the American Association for Cancer Research 07// 2018;24(14):3247-3252  2018 07/ | 13. No quantified outcome measure reported (clinical response / prognosis / Progression free survival / survival etc.) in relation to a sleep measure | |
| #3941 - Chan 2008  Chan, Joseph S; Beer, Tomasz M; Quinn, David I; Pinski, Jacek K; Garzotto, Mark; Sokoloff, Mitchell; Dehaze, Daniel R; Ryan, Christopher W  A phase II study of high-dose calcitriol combined with mitoxantrone and prednisone for androgen-independent prostate cancer.  BJU international 12// 2008;102(11):1601-1606  2008 12// | 13. No quantified outcome measure reported (clinical response / prognosis / Progression free survival / survival etc.) in relation to a sleep measure | |
| #5106 - Chen 2002  Chen, Fu-xing; Liu, Jun-quan; Zhang, Nan-zheng; Gong, Xin-jian; Zhang, Guo-long; Xu, Yong-mao; Zhou, Zhong-hai; Wang, Tao; Huang, Jian  [Clinical observation on adoptive immunotherapy with autologous cytokine-induced killer cells for advanced malignant tumor] | 2. No full text to be found | |
| #4138 - Chen 2011  Chen, Xing-Gui; Huang, He; Tian, Ying; Guo, Cheng-Cheng; Liang, Chao-Yong; Gong, Yao-Ling; Zou, Ben-Yan; Cai, Rui-Qing; Lin, Tong-Yu  Cyclosporine, prednisone, and high-dose immunoglobulin treatment of angioimmunoblastic T-cell lymphoma refractory to prior CHOP or CHOP-like regimen.  Chinese journal of cancer 10// 2011;30(10):731-738  2011 10// | 13. No quantified outcome measure reported (clinical response / prognosis / Progression free survival / survival etc.) in relation to a sleep measure | |
| #4143 - Chung 2010  Chung, Man Ki; Son, Young-Ik; Cho, Jae Keun; So, Yoon Kyoung; Woo, Seung Hoon; Jeong, Han-Sin; Baek, Chung-Hwan  Therapeutic options in patients with early T stage and advanced N stage of tonsillar squamous cell carcinomas.  Otolaryngology--head and neck surgery : official journal of American Academy of Otolaryngology-Head and Neck Surgery 12// 2010;143(6):808-814  2010 12// | 13. No quantified outcome measure reported (clinical response / prognosis / Progression free survival / survival etc.) in relation to a sleep measure | |
| #2725 - Coens 2017  Coens, Corneel; Suciu, Stefan; Chiarion-Sileni, Vanna; Grob, Jean-Jacques; Dummer, Reinhard; Wolchok, Jedd D; Schmidt, Henrik; Hamid, Omid; Robert, Caroline; Ascierto, Paolo A; Richards, Jon M; Lebbé, Celeste; Ferraresi, Virginia; Smylie, Michael; Weber, Jeffrey S; Maio, Michele; Bottomley, Andrew; Kotapati, Srividya; de Pril, Veerle; Testori, Alessandro; Eggermont, Alexander M M  Health-related quality of life with adjuvant ipilimumab versus placebo after complete resection of high-risk stage III melanoma (EORTC 18071): secondary outcomes of a multinational, randomised, double-blind, phase 3 trial.  The Lancet. Oncology 03// 2017;18(3):393-403  2017 03// | 13. No quantified outcome measure reported (clinical response / prognosis / Progression free survival / survival etc.) in relation to a sleep measure | |
| #4333 - Dahiya 2016  Dahiya, Neha; Acharya, Anita S; Bachani, Damodar; Sharma, Dn; Gupta, Subhash; Haresh, Kp; Rath, Gk  Quality of Life of Patients with Advanced Cervical Cancer before and after Chemoradiotherapy.  Asian Pacific journal of cancer prevention : APJCP // 2016;17(7):3095-3099  2016 // | 13. No quantified outcome measure reported (clinical response / prognosis / Progression free survival / survival etc.) in relation to a sleep measure | |
| #855 - Dieras 2017  Dieras, V; Robson, ME; Palacova, M; Marcom, PK; Jager, A; Bondarenko, I; Citrin, D; Campone, M; Telli, ML; Domchek, SM; et al.  Efficacy and tolerability of veliparib (V; ABT-888) in combination with carboplatin (C) and paclitaxel (P) vs placebo (Plc)+C/P in patients (PTS) with BRCA1 or BRCA2 mutations and metastatic breast cancer: a randomized, phase 2 study HS Han  2017; 77( 4 Supplement 1) (no pagination):  2017 | 4. Not primary research (empirical research study | |
| #3683 - Dómine 2019  Dómine, Manuel; Massuti, Bartomeu; Puente, Javier; Calles, Antonio; Esteban, Emilio; Triguboff, Eduardo; Afonzo, Yashmin Silvana; Gironés, Regina; Aparisi, Francisco; Oramas, Juana  Observational Prospective Study to Determine the Evolution of the Symptomatic Profile of Metastatic Non-Small Cell Lung Cancer (NSCLC) Patients and Its Relation to the Control of the Disease.  Advances in therapy 06// 2019;36(6):1497-1508  2019 06// | 13. No quantified outcome measure reported (clinical response / prognosis / Progression free survival / survival etc.) in relation to a sleep measure | |
| #2785 - Dummer 2014  Dummer, R; Duvic, M; Scarisbrick, J; Olsen, E A; Rozati, S; Eggmann, N; Goldinger, S M; Hutchinson, K; Geskin, L; Illidge, T M; Giuliano, E; Elder, J; Kim, Y H  Final results of a multicenter phase II study of the purine nucleoside phosphorylase (PNP) inhibitor forodesine in patients with advanced cutaneous T-cell lymphomas (CTCL) (Mycosis fungoides and Sézary syndrome).  Annals of oncology : official journal of the European Society for Medical Oncology 09// 2014;25(9):1807-1812  2014 09/ | 13. No quantified outcome measure reported (clinical response / prognosis / Progression free survival / survival etc.) in relation to a sleep measure | |
| #4393 - Dutcher 2000  Dutcher, J P; Logan, T; Gordon, M; Sosman, J; Weiss, G; Margolin, K; Plasse, T; Mier, J; Lotze, M; Clark, J; Atkins, M  Phase II trial of interleukin 2, interferon alpha, and 5-fluorouracil in metastatic renal cell cancer: a cytokine working group study.  Clinical cancer research : an official journal of the American Association for Cancer Research 09// 2000;6(9):3442-3450  2000 09// | 13. No quantified outcome measure reported (clinical response / prognosis / Progression free survival / survival etc.) in relation to a sleep measure | |
| #3421 - Eisen 2000  Eisen, T; Boshoff, C; Mak, I; Sapunar, F; Vaughan, M M; Pyle, L; Johnston, S R; Ahern, R; Smith, I E; Gore, M E  Continuous low dose Thalidomide: a phase II study in advanced melanoma, renal cell, ovarian and breast cancer.  British journal of cancer 02// 2000;82(4):812-817  2000 02// | 13. No quantified outcome measure reported (clinical response / prognosis / Progression free survival / survival etc.) in relation to a sleep measure | |
| #410 - Engert 2017  Engert, A; Taylor, F; Bennett, B; Chen, C; Cocks, K; McDonald, J; Mann, E; Sacchi, M; Cella, D  Effect of nivolumab on patient-reported outcomes in patients with relapsed/refractory classical hodgkin lymphoma after autologous transplantation: results from the multicohort phase 2 checkmate 205 study  2017; 130():  2017 | 4. Not primary research (empirical research study) | |
| #677 - Fagotti 2016  Fagotti, A; Ferrandina, G; Vizzielli, G; Fanfani, F; Gallotta, V; Chiantera, V; Costantini, B; Margariti, PA; Gueli Alletti, S; Cosentino, F; et al.  Phase III randomised clinical trial comparing primary surgery versus neoadjuvant chemotherapy in advanced epithelial ovarian cancer with high tumour load (SCORPION trial): final analysis of peri-operative outcome  2016; 59(): 22‐33  201 | 13. No quantified outcome measure reported (clinical response / prognosis / Progression free survival / survival etc.) in relation to a sleep measure | |
| #726 - Fernando 2011  Fernando, I; Bowden, SJ; Fox, RP; Grieve, R; Brunt, AM; Agrawal, RK; Ritchie, D; Simmonds, P; Bishop, J; Rea, DW  Effect of synchronous chemo-radiation on quality of life: results from the SECRAB trial (ISRCTN: 84214355) presented on behalf of the SECRAB steering committee  2011; 47(): S366  2011 | 4. Not primary research (empirical research study) | |
| #4185 - Focan 2003  Focan, C; Mormont, C; Moeneclaye, N; Focan-Henrard, D; Waterhouse, J  [Evaluation of rest-activity rhythms in advanced non-small-cell lung cancer (NSCLC) patients. Importance of corticotherapy?].  Pathologie-biologie 06// 2003;51(4):206-207  2003 06// | 3. Not English language | |
| #4754 - Fosså 1990  Fosså, S D; Sager, E M; Hosbach, G; Waehre, H; Ous, S  Cisplatin and medium dose methotrexate in advanced transitional cell carcinoma of the urinary tract.  Scandinavian journal of urology and nephrology // 1990;24(3):199-204  1990 // | 13. No quantified outcome measure reported (clinical response / prognosis / Progression free survival / survival etc.) in relation to a sleep measure | |
| #5223 - Gelibter 2005  Gelibter, Alain; Ceribelli, Anna; Pollera, Camillo F; Milella, Michele; Moscetti, Luca; Sperduti, Isabella; Cognetti, Francesco  Impact of gefitinib ('Iressa') treatment on the quality of life of patients with advanced non-small-cell lung cancer.  Journal of cancer research and clinical oncology 12// 2005;131(12):783-788  2005 12// | 13. No quantified outcome measure reported (clinical response / prognosis / Progression free survival / survival etc.) in relation to a sleep measure | |
| #575 - Goldenberg 1999  Goldenberg, MM  Trastuzumab, a recombinant DNA-derived humanized monoclonal antibody, a novel agent for the treatment of metastatic breast cancer  1999; 21( 2): 309‐318  1999 | 4. Not primary research (empirical research study) | |
| #4796 - Gonella 2010  Gonella, S  [Sleep-wake cycle in chemotherapy patients: a retrospective study].  Minerva medica 06// 2010;101(3):135-147  2010 06// | 3. Not English language | |
| #1122 - Gourgou-Bourgade 2013  Gourgou-Bourgade, S; Bascoul-Mollevi, C; Desseigne, F; Ychou, M; Bouché, O; Guimbaud, R; Bécouarn, Y; Adenis, A; Raoul, JL; Boige, V; et al.  Impact of FOLFIRINOX compared with gemcitabine on quality of life in patients with metastatic pancreatic cancer: results from the PRODIGE 4/ACCORD 11 randomized trial  2013; 31( 1): 23‐29  2013 | 13. No quantified outcome measure reported (clinical response / prognosis / Progression free survival / survival etc.) in relation to a sleep measure | |
| #1138 - Greimel 2013  Greimel, E; Kristensen, GB; van der Burg, ME; Coronado, P; Rustin, G; del Rio, AS; Reed, NS; Nordal, RR; Coens, C; Vergote, I  Quality of life of advanced ovarian cancer patients in the randomized phase III study comparing primary debulking surgery versus neo-adjuvant chemotherapy  2013; 131( 2): 437‐444  2013 | 13. No quantified outcome measure reported (clinical response / prognosis / Progression free survival / survival etc.) in relation to a sleep measure | |
| #4509 - Gridelli 2007  Gridelli, Cesare; Gallo, Ciro; Ceribelli, Anna; Gebbia, Vittorio; Gamucci, Teresa; Ciardiello, Fortunato; Carozza, Francesco; Favaretto, Adolfo; Daniele, Bruno; Galetta, Domenico; Barbera, Santi; Rosetti, Francesco; Rossi, Antonio; Maione, Paolo; Cognetti, Francesco; Testa, Antonio; Di Maio, Massimo; Morabito, Alessandro; Perrone, Francesco  Factorial phase III randomised trial of rofecoxib and prolonged constant infusion of gemcitabine in advanced non-small-cell lung cancer: the GEmcitabine-COxib in NSCLC (GECO) study.  The Lancet. Oncology 06// 2007;8(6):500-512  2007 06// | 13. No quantified outcome measure reported (clinical response / prognosis / Progression free survival / survival etc.) in relation to a sleep measure | |
| #3425 - Grob 2014  Grob, J-J; Amonkar, M M; Martin-Algarra, S; Demidov, L V; Goodman, V; Grotzinger, K; Haney, P; Kämpgen, E; Karaszewska, B; Mauch, C; Miller, W H Jr; Millward, M; Mirakhur, B; Rutkowski, P; Chiarion-Sileni, V; Swann, S; Hauschild, A  Patient perception of the benefit of a BRAF inhibitor in metastatic melanoma: quality-of-life analyses of the BREAK-3 study comparing dabrafenib with dacarbazine.  Annals of oncology : official journal of the European Society for Medical Oncology 07// 2014;25(7):1428-1436  2014 07// | 13. No quantified outcome measure reported (clinical response / prognosis / Progression free survival / survival etc.) in relation to a sleep measure | |
| #345 - Gu 2016  Gu, L P; Ye, X Y; Xu, Y H; Hou, W X; Li, J Q; Yao, Z L; Bi, L; Lu, S; Xu, L; Chen, Z W  A double-bind and randomized controlled clinical trial of traditional Chinese medicine combined with adjuvant chemotherapy for early stage non-small cell lung cancer  2016; 28( 6): 394‐8, 403  2016 | 3. Not English language | |
| #230 - Gudipudi 2014  Gudipudi, GK; Del Priore, G; Alluri, KR  Comparing intensity-modulated radiotherapy and conventional external beam radiotherapy in cervical cancer  2014; 133(): 13  2014 | 4. Not primary research (empirical research study) | |
| #1184 - Guo 2013  Guo, Z; Tang, HY; Li, H; Tan, SK; Feng, KH; Huang, YC; Bu, Q; Jiang, W  The benefits of psychosocial interventions for cancer patients undergoing radiotherapy  2013; 11(): 121  2013 | 13. No quantified outcome measure reported (clinical response / prognosis / Progression free survival / survival etc.) in relation to a sleep measure | |
| #4376 - Gwak 2014  Gwak, Ho-Shin; Joo, Jungnam; Shin, Sang-Hoon; Yoo, Heon; Han, Ji-Youn; Kim, Heung Tae; Yun, Tak; Ro, Jungsil; Lee, Jin Soo; Lee, Seung Hoon  Ventriculolumbar perfusion chemotherapy with methotrexate for treating leptomeningeal carcinomatosis: a Phase II Study.  The oncologist 10// 2014;19(10):1044-1045  2014 10// | 4. Not primary research (empirical research study) | |
| #4923 - Haddock 2007  Haddock, Michael G; Swaminathan, Revathi; Foster, Nathan R; Hauge, Mark D; Martenson, James A; Camoriano, John K; Stella, Philip J; Tenglin, Richard C; Schaefer, Paul L; Moore, Dennis F Jr; Alberts, Steven R  Gemcitabine, cisplatin, and radiotherapy for patients with locally advanced pancreatic adenocarcinoma: results of the North Central Cancer Treatment Group Phase II Study N9942.  Journal of clinical oncology : official journal of the American Society of Clinical Oncology 06// 2007;25(18):2567-2572  2007 06// | 13. No quantified outcome measure reported (clinical response / prognosis / Progression free survival / survival etc.) in relation to a sleep measure | |
| #913 - Han 2017  Han, HS; Dieras, V; Robson, ME; Palacova, M; Marcom, PK; Jager, A; Bondarenko, I; Citrin, D; Campone, M; Telli, ML; et al.  Efficacy and tolerability of veliparib (V; ABT-888) in combination with carboplatin (C) and paclitaxel (P) vs placebo (Plc)+C/P in patients (pts) with BRCA1 orBRCA2 mutations and metastatic breast cancer: a randomized, phase 2 study  2017; 77( 4):  2017 | 4. Not primary research (empirical research study | |
| #4667 - Hassler 2015  Hassler, Marco Ronald; Sax, Cornelia; Flechl, Birgit; Ackerl, Michael; Preusser, Matthias; Hainfellner, Johannes Andreas; Woehrer, Adelheid; Dieckmann, Karin Ute; Rössler, Karl; Prayer, Daniela; Marosi, Christine  Thalidomide as palliative treatment in patients with advanced secondary glioblastoma.  Oncology // 2015;88(3):173-179  2015 // | 13. No quantified outcome measure reported (clinical response / prognosis / Progression free survival / survival etc.) in relation to a sleep measure | |
| #4467 - Herrmann 2009  Herrmann, Edwin; Gerss, Joachim; Bierer, Stefan; Köpke, Thomas; Bolenz, Christian; Hertle, Lothar; Wülfing, Christian  Pre-treatment global quality of health predicts progression free survival in metastatic kidney cancer patients treated with sorafenib or sunitinib.  Journal of cancer research and clinical oncology 01// 2009;135(1):61-67  2009 01// | 13. No quantified outcome measure reported (clinical response / prognosis / Progression free survival / survival etc.) in relation to a sleep measure | |
| #891 - Hilgenfeld 1997  Hilgenfeld, RU; Mansmann, U; Guggenmoos-Holzmann, I; Thiel1, E; Kreuser, ED  Quality of life (QL) is a prognostic factor (PF) for survival in patients with advanced colorectal cancer (CRC)  1997;(): Abstract #760  199 | 4. Not primary research (empirical research study) | |
| #540 - Hjorth 2012  Hjorth, M; Hjertner, Ø; Knudsen, LM; Gulbrandsen, N; Holmberg, E; Pedersen, PT; Andersen, NF; Andréasson, B; Billström, R; Carlson, K; et al.  Thalidomide and dexamethasone vs. bortezomib and dexamethasone for melphalan refractory myeloma: a randomized study  2012; 88( 6): 485‐496  2012 | 13. No quantified outcome measure reported (clinical response / prognosis / Progression free survival / survival etc.) in relation to a sleep measure | |
| #976 - Hlubocky 2017  Hlubocky, FJ; Sher, T; Cella, D; Yap, BJ; Ratain, MJ; Peppercorn, J; Daugherty, C  The impact of sleep disturbances (SD) on quality of life, psychological morbidity, and survival of advanced cancer patients (ACP) and caregivers (CG)  2017; 35( 15 Supplement 1) (no pagination):  2017 | 4. Not primary research (empirical research study) | |
| #907 - Jagannath 2006  Jagannath, S; Richardson, PG; Barlogie, B; Berenson, JR; Singhal, S; Irwin, D; Srkalovic, G; Schenkein, DP; Esseltine, DL; Anderson, KC  Bortezomib in combination with dexamethasone for the treatment of patients with relapsed and/or refractory multiple myeloma with less than optimal response to bortezomib alone  2006; 91( 7): 929‐934  2006 | 13. No quantified outcome measure reported (clinical response / prognosis / Progression free survival / survival etc.) in relation to a sleep measure | |
| #5275 - Kozachik 2008  Kozachik, Sharon L; Bandeen-Roche, Karen  Predictors of patterns of pain, fatigue, and insomnia during the first year after a cancer diagnosis in the elderly.  Cancer nursing // 2008;31(5):334-344  2008 // | 13. No quantified outcome measure reported (clinical response / prognosis / Progression free survival / survival etc.) in relation to a sleep measure | |
| #1133 - Lee 2009  Lee, SM; Rudd, R; Woll, PJ; Ottensmeier, C; Gilligan, D; Price, A; Spiro, S; Gower, N; Jitlal, M; Hackshaw, A  Randomized double-blind placebo-controlled trial of thalidomide in combination with gemcitabine and Carboplatin in advanced non-small-cell lung cancer  2009; 27( 31): 5248‐5254  2009 | 13. No quantified outcome measure reported (clinical response / prognosis / Progression free survival / survival etc.) in relation to a sleep measure | |
| #792 - Lee 2009  Lee, SM; Woll, PJ; Rudd, R; Ferry, D; O'Brien, M; Middleton, G; Spiro, S; James, L; Ali, K; Jitlal, M; et al.  Anti-angiogenic therapy using thalidomide combined with chemotherapy in small cell lung cancer: a randomized, double-blind, placebo-controlled trial  2009; 101( 15): 1049‐1057  2009 | 13. No quantified outcome measure reported (clinical response / prognosis / Progression free survival / survival etc.) in relation to a sleep measure | |
| #3711 - Lee 2008  Lee, Stephanie J; Richardson, Paul G; Sonneveld, Pieter; Schuster, Michael W; Irwin, David; San Miguel, Jesús-F; Crawford, Bruce; Massaro, Joseph; Dhawan, Ravinder; Gupta, Sanjay; Anderson, Kenneth C  Bortezomib is associated with better health-related quality of life than high-dose dexamethasone in patients with relapsed multiple myeloma: results from the APEX study.  British journal of haematology 11// 2008;143(4):511-519  2008 11// | 13. No quantified outcome measure reported (clinical response / prognosis / Progression free survival / survival etc.) in relation to a sleep measure | |
| #506 - Martin 2015  Martin, LS; Garcia, JLS; Guerra, SA; Hernandez, ZG; Abreu, IR  Evaluation of the antitumor effect of nimotuzumab combined with radiochemotherapy in treatment of esophageal tumors  2015; 49( 2):  2015 | 3. Not English language | |
| #3913 - Minniti 2013  Minniti, Giuseppe; Scaringi, Claudia; Baldoni, Alessandra; Lanzetta, Gaetano; De Sanctis, Vitaliana; Esposito, Vincenzo; Enrici, Riccardo Maurizi  Health-related quality of life in elderly patients with newly diagnosed glioblastoma treated with short-course radiation therapy plus concomitant and adjuvant temozolomide.  International journal of radiation oncology, biology, physics 06// 2013;86(2):285-291  2013 06// | 13. No quantified outcome measure reported (clinical response / prognosis / Progression free survival / survival etc.) in relation to a sleep measure | |
| #774 - Nakano 1999  Nakano, H; Namatame, K; Nemoto, H; Motohashi, H; Nishiyama, K; Kumada, K  A multi-institutional prospective study of lentinan in advanced gastric cancer patients with unresectable and recurrent diseases: effect on prolongation of survival and improvement of quality of life. Kanagawa Lentinan Research Group  1999; 46( 28): 2662‐2668  1999 | 2. No full text to be found | |
| #4856 - Narayanan 2013  Narayanan, Govindarajan; Barbery, Katuzka; Suthar, Rekha; Guerrero, Gabriella; Arora, Geetika  Transarterial chemoembolization using DEBIRI for treatment of hepatic metastases from colorectal cancer.  Anticancer research 05// 2013;33(5):2077-2083  2013 05// | 13. No quantified outcome measure reported (clinical response / prognosis / Progression free survival / survival etc.) in relation to a sleep measure | |
| #4218 - Nasta 2003  Nasta, Sunita Dwivedy; Hoff, Paulo M; George, Christopher S; Neubauer, Marcus; Cohen, Steven C; Abbruzzese, James; Winn, Rodger; Pazdur, Richard M  Phase II study of MGI-114 administered intravenously for 5 days every 28 days to patients with metastatic colorectal cancer.  American journal of clinical oncology 04// 2003;26(2):132-134  2003 04// | 13. No quantified outcome measure reported (clinical response / prognosis / Progression free survival / survival etc.) in relation to a sleep measure | |
| #323 - Nct 2018  Nct,  12-week Exercise Intervention Program Versus Observation in Early Stage Breast Cancer Patients on the Impact on Mental Health, Quality of Life and Immune Markers  2018;():  2018 | 4. Not primary research (empirical research study) | |
| #1252 - NCT03518957 2018  NCT03518957,  12-week Exercise Intervention Program Versus Observation in Early Stage Breast Cancer Patients on the Impact on Mental Health, Quality of Life and Immune Markers  2018;():  2018 | 1. Dublicate | |
| #4983 - Niu 2014  Niu, Jingxiu; Ren, Yanjie; Zhang, Tianyu; Yang, Xuejing; Zhu, Wei; Zhu, Hui; Li, Jing; Li, Jiali; Pang, Yan  Retrospective comparative study of the effects of dendritic cell vaccine and cytokine-induced killer cell immunotherapy with that of chemotherapy alone and in combination for colorectal cancer.  BioMed research international // 2014;2014():214727-214727  2014 // | 13. No quantified outcome measure reported (clinical response / prognosis / Progression free survival / survival etc.) in relation to a sleep measure | |
| #4042 - Norsa 2006  Norsa, Achille; Martino, Vincenzo  Somatostatin, retinoids, melatonin, vitamin D, bromocriptine, and cyclophosphamide in advanced non-small-cell lung cancer patients with low performance status.  Cancer biotherapy & radiopharmaceuticals 02// 2006;21(1):68-73  2006 02// | 13. No quantified outcome measure reported (clinical response / prognosis / Progression free survival / survival etc.) in relation to a sleep measure | |
| #633 - O'Brien 2000  O'Brien, ME; Saini, A; Smith, IE; Webb, A; Gregory, K; Mendes, R; Ryan, C; Priest, K; Bromelow, KV; Palmer, RD; et al.  A randomized phase II study of SRL172 (Mycobacterium vaccae) combined with chemotherapy in patients with advanced inoperable non-small-cell lung cancer and mesothelioma  2000; 83( 7): 853‐857  2000 | 13. No quantified outcome measure reported (clinical response / prognosis / Progression free survival / survival etc.) in relation to a sleep measure | |
| #416 - O'Hara 2017  O'Hara, MH; Karasic, TB; Vasilevskaya, I; Redlinger, M; Loaiza-Bonilla, A; Teitelbaum, UR; Giantonio, BJ; Damjanov, N; Reiss, KA; Rosen, MA; et al.  Phase II trial of the autophagy inhibitor hydroxychloroquine with FOLFOX and bevacizumab in front line treatment of metastatic colorectal cancer  2017; 35( 15):  2017 | 4. Not primary research (empirical research study) | |
| #721 - Paccagnella 2004  Paccagnella, A; Favaretto, A; Oniga, F; Barbieri, F; Ceresoli, G; Torri, W; Villa, E; Verusio, C; Cetto, GL; Santo, A; et al.  Cisplatin versus carboplatin in combination with mitomycin and vinblastine in advanced non small cell lung cancer. A multicenter, randomized phase III trial  2004; 43( 1): 83‐91  2004 | 13. No quantified outcome measure reported (clinical response / prognosis / Progression free survival / survival etc.) in relation to a sleep measure | |
| #4647 - Park 2009  Park, Hee Chul; Janjan, Nora A; Mendoza, Tito R; Lin, Edward H; Vadhan-Raj, Saroj; Hundal, Mandeep; Zhang, Yiqun; Delclos, Marc E; Crane, Christopher H; Das, Prajnan; Wang, Xin Shelley; Cleeland, Charles S; Krishnan, Sunil  Temporal patterns of fatigue predict pathologic response in patients treated with preoperative chemoradiation therapy for rectal cancer.  International journal of radiation oncology, biology, physics 11// 2009;75(3):775-781  2009 11/ | 13. No quantified outcome measure reported (clinical response / prognosis / Progression free survival / survival etc.) in relation to a sleep measure | |
| #3909 - Popiela 2001  Popiela, T; Kulig, J; Hanisch, J; Bock, P R  Influence of a complementary treatment with oral enzymes on patients with colorectal cancers--an epidemiological retrolective cohort study.  Cancer chemotherapy and pharmacology 07// 2001;47 Suppl():S55-63  2001 07// | 13. No quantified outcome measure reported (clinical response / prognosis / Progression free survival / survival etc.) in relation to a sleep measure | |
| #3039 - Pyrhönen 1990  Pyrhönen, S; Valavaara, R; Heikkinen, M; Rissanen, P; Blanco, G; Nordman, E; Holsti, L R; Hajba, A  Treatment of advanced breast cancer with 20 mg toremifene, a phase II study. Preliminary communication.  Journal of steroid biochemistry 06// 1990;36(3):227-228  1990 06// | 13. No quantified outcome measure reported (clinical response / prognosis / Progression free survival / survival etc.) in relation to a sleep measure | |
| #927 - Robson 2018  Robson, M; Hettle, R; Degboe, A; Saunders, O; Cain, T; Kilvert, H; Johnson, H  Estimating the health state utility of patients with HER2-gBRCA + metastatic breast cancer treated with olaparib or chemotherapy via a mapping analysis of EORTC QLQ-C30 data collected in the olympiad clinical trial  2018; 21(): S12‐  2018 | 4. Not primary research (empirical research study) | |
| #3961 - Schilder 2012  Schilder, Russell J; Brady, William E; Lankes, Heather A; Fiorica, James V; Shahin, Mark S; Zhou, Xun C; Mannel, Robert S; Pathak, Harsh B; Hu, Wei; Alpaugh, R Katherine; Sood, Anil K; Godwin, Andrew K  Phase II evaluation of dasatinib in the treatment of recurrent or persistent epithelial ovarian or primary peritoneal carcinoma: a Gynecologic Oncology Group study.  Gynecologic oncology 10// 2012;127(1):70-74  2012 10// | 13. No quantified outcome measure reported (clinical response / prognosis / Progression free survival / survival etc.) in relation to a sleep measure | |
| #517 - Shaik 2018  Shaik, F; Uldrick, TS; Esterhuizen, T; Mosam, A  Health-Related Quality of Life in Patients Treated With Antiretroviral Therapy Only Versus Chemotherapy and Antiretroviral Therapy for HIV-Associated Kaposi Sarcoma: a Randomized Control Trial  2018; 4(): 1‐9  2018 | 13. No quantified outcome measure reported (clinical response / prognosis / Progression free survival / survival etc.) in relation to a sleep measure | |
| #4527 - Stobäus 2015  Stobäus, Nicole; Müller, Manfred J; Küpferling, Susanne; Schulzke, Jörg-Dieter; Norman, Kristina  Low Recent Protein Intake Predicts Cancer-Related Fatigue and Increased Mortality in Patients with Advanced Tumor Disease Undergoing Chemotherapy.  Nutrition and cancer // 2015;67(5):818-824  2015 // | 13. No quantified outcome measure reported (clinical response / prognosis / Progression free survival / survival etc.) in relation to a sleep measure | |
| #547 - Tamura 2000  Tamura, H; Kojima, M; Kobayashi, H; Ando, J; Oka, S; Fujisaki, M; Wada, N; Imoto, S; Ikeda, T  Randomized comparative study of CMF (cyclophosphamide, methotrexate and 5-fluorouracil) and UFT-tamoxifen regimens as adjuvant chemotherapy after surgery for breast cancer: tochigi Prefectural Study Group for Post-Breast Cancer Adjuvant Chemotherapy  2000; 27( 7): 993‐1002  2000 | 3. Not English language | |
| #904 - Tanaka 2012  Tanaka, H; Genma, A; Sakai, H; Nishio, M; Inoue, A; Okamoto, H; Takiguchi, Y; Isobe, H; Kunitoh, H; Kubota, K; et al.  Randomized phase iii trial of S-1 plus cisplatin versus docetaxel plus cisplatin for advanced nonsmall-cell lung cancer (TCOG0701)  2012; 7( 11 SUPPL. 5): S445  2012 | 4. Not primary research (empirical research study) | |
| #938 - Topp 2016  Topp, MS; Zimmerman, Z; Cannell, P; Dombret, H; Maertens, J; Schuh, AC; Franklin, J; Nie, K; Cong, Z  Health-related quality of life (HRQoL) of blinatumomab versus standard of care (SOC) chemotherapy in patients with relaspsed or refractory philadelphia negative B-Cell precursor acute lymphoblastic leukemia in a randomized, open-label phase 3 study (TOWE  2016; 128( 22):  2016 | 4. Not primary research (empirical research study | |
| #631 - Topp 2018  Topp, MS; Zimmerman, Z; Cannell, P; Dombret, H; Maertens, J; Stein, A; Franklin, J; Tran, Q; Cong, Z; Schuh, AC  Health-related quality of life in adults with relapsed/refractory acute lymphoblastic leukemia treated with blinatumomab  2018; 131( 26): 2906‐2914  2018 | 13. No quantified outcome measure reported (clinical response / prognosis / Progression free survival / survival etc.) in relation to a sleep measure | |
| #4219 - vanAndel 2003  van Andel, G; Fernandez de Moral, P; Caris, C T M; Carpentier, P; Wils, J; de Bruin, M J F M; Witjes, J A; Debruyne, F M J; Witjes, W P J  A randomized study comparing epirubicin in a 4-weekly versus a weekly intravenous regimen in patients with metastatic, hormone resistant, prostatic carcinoma: effects on health related quality of life.  World journal of urology 08// 2003;21(3):177-182  2003 08// | 13. No quantified outcome measure reported (clinical response / prognosis / Progression free survival / survival etc.) in relation to a sleep measure | |
| #3379 - Vergote 1992  Vergote, I; Himmelmann, A; Frankendal, B; Scheistrøen, M; Vlachos, K; Tropé, C  Hexamethylmelamine as second-line therapy in platin-resistant ovarian cancer.  Gynecologic oncology 12// 1992;47(3):282-286  1992 12// | 13. No quantified outcome measure reported (clinical response / prognosis / Progression free survival / survival etc.) in relation to a sleep measure | |
| #4155 - Vinik 2016  Vinik, Aaron; Bottomley, Andrew; Korytowsky, Beata; Bang, Yung-Jue; Raoul, Jean-Luc; Valle, Juan W; Metrakos, Peter; Hörsch, Dieter; Mundayat, Rajiv; Reisman, Arlene; Wang, Zhixiao; Chao, Richard C; Raymond, Eric  Patient-Reported Outcomes and Quality of Life with Sunitinib Versus Placebo for Pancreatic Neuroendocrine Tumors: Results From an International Phase III Trial.  Targeted oncology 12// 2016;11(6):815-824  2016 12// | 13. No quantified outcome measure reported (clinical response / prognosis / Progression free survival / survival etc.) in relation to a sleep measure | |
| #906 - Wang 2013  Wang, L; Sun, Z X; Feng, G Q; Ma, S J  Effect of maintenance treatment by traditional Chinese medicine syndrome differentiation on life quality and progress-free survival of patients with advanced non-small cell lung cancer after chemotherapy  2013; 19( 13): 319‐322  2013 | 8. Review | |
| #2271 - Wang 2014  Wang, Shiyong; Zhang, Hui; Liu, Chang; Jiao, Xue; Liu, Dijie; DU, Weili; He, Ying; Zhang, Zhe; Wu, Xiuyan; Wang, Jialing; Liang, Chunyan; Zhang, Lu; Liu, Shu  Human leukocyte antigen-haploidentical donor-derived cytokine-induced killer cells are safe and prolong the survival of patients with advanced non-small cell lung cancer.  Oncology letters 12// 2014;8(6):2727-2733  2014 12// | 13. No quantified outcome measure reported (clinical response / prognosis / Progression free survival / survival etc.) in relation to a sleep measure | |
| #3014 - Watanabe 2002  Watanabe, Toru; Sano, Muneaki; Toi, Masakazu; Saeki, Toshiaki; Kanda, Kazuhiro; Miura, Shigeto; Inaji, Hideo; Sono, Hiroshi; Saeki, Hideyuki; Nishimura, Reiki; Fujita, Yoshie  [Late phase II study of exemestane in postmenopausal patients with breast cancer resistant to anti-estrogenic agents].  Gan to kagaku ryoho. Cancer & chemotherapy 07// 2002;29(7):1211-1221  2002 07// | 3. Not English language | |
| #3659 - Wymenga 1999  Wymenga, A N; Eriksson, B; Salmela, P I; Jacobsen, M B; Van Cutsem, E J; Fiasse, R H; Välimäki, M J; Renstrup, J; de Vries, E G; Oberg, K E  Efficacy and safety of prolonged-release lanreotide in patients with gastrointestinal neuroendocrine tumors and hormone-related symptoms.  Journal of clinical oncology : official journal of the American Society of Clinical Oncology 04// 1999;17(4):1111-1111  1999 04// | 13. No quantified outcome measure reported (clinical response / prognosis / Progression free survival / survival etc.) in relation to a sleep measure | |
| #870 - Yang 2016  Yang, J; Gu, G; Wang, X; Li, X; Zhan, Y; Liu, C  Clinical effectiveness of autologous tumor antigen-pulsed dendritic cell-cytokine induced killer cells combined with chemotherapy in treatment of advanced lung adenocarcinoma  2016; 23( 1): 83‐88  2016 | 3. Not English language | |
| #4508 - Yang 2019  Yang, Yang; Wu, Xiuwei; Li, Fanfan; Wang, Nianfei; Zhang, Mingjun; Sun, Tong; Chen, Zhendong  Evaluation of efficacy and safety of apatinib treatment in advanced gastric cancer.  Journal of cancer research and therapeutics // 2019;15(2):365-369  2019 // | 13. No quantified outcome measure reported (clinical response / prognosis / Progression free survival / survival etc.) in relation to a sleep measure | |
| #2915 - Zhang 2016  Zhang, Lihong; Zhu, Wei; Li, Jiali; Yang, Xuejing; Ren, Yanjie; Niu, Jingxiu; Pang, Yan  Clinical outcome of immunotherapy with dendritic cell vaccine and cytokine-induced killer cell therapy in hepatobiliary and pancreatic cancer.  Molecular and clinical oncology 01// 2016;4(1):129-133  2016 01// | 13. No quantified outcome measure reported (clinical response / prognosis / Progression free survival / survival etc.) in relation to a sleep measure | |
| #5339 - Zhu 2014  Zhu, H; Yang, X; Li, J; Ren, Y; Zhang, T; Zhang, C; Zhang, J; Pang, Y  Immune response, safety, and survival and quality of life outcomes for advanced colorectal cancer patients treated with dendritic cell vaccine and cytokine-induced killer cell therapy  BioMed Research International // 2014;2014():  2014 // | 13. No quantified outcome measure reported (clinical response / prognosis / Progression free survival / survival etc.) in relation to a sleep measure | |
| #576  Comparison of health-related quality of life with epirubicin, cisplatin plus 5-fluorouracil and docetaxel, cisplatin plus 5-fluorouracil chemotherapy regimens as first-line systemic therapy in locally advanced inoperable or metastatic gastric or gastro-e  2018; 7( 1): 11‐15  2018 | 13. No quantified outcome measure reported (clinical response / prognosis / Progression free survival / survival etc.) in relation to a sleep measure | |
| **List of criteria for exclusion - hierarchically displayed:**  1. Dublicate  2. No full text to be found  3. Not English language  4. Not primary research (E.g.; conference abstract, study protocol)  5. Animal/cell study  6. Participants were not adults  7. Participants were not diagnosed with cancer  8. Review  9. Phase 1 clinical trails  10. Single cases/case studies  11. Participants did not receive oncological treatment  12. No quantified sleep measure reported  13. No quantified outcome measure reported (clinical response / prognosis / Progression free survival / survival etc.) in relation to a sleep measure  14. Treatment was transplantation  15. No assessment of sleep measure immediately prior to or during oncological treatment.  16. Oncological treatment was not initiated before the outcome measure was assessed (Clinical response, Time to progression, OR survival, OR prognosis).  17. Paper derived from study with multiple published report, where a more detailed report from this study is already included.  18. Sleep measure was not assessed post-diagnosis and pre-outcome-measure (clinical response / prognosis / Progression free survival / survival) | |  |
