# Supplementary material for: Sleep During Oncological Treatment – A Systematic Review and Meta-Analysis of Associations With Treatment Response, Time to Progression and Survival
Source: Front Neurosci. 2022 Apr 19;16:817837. doi: 10.3389/fnins.2022.817837 (PMC9063131; doi:10.3389/fnins.2022.817837)
Supplement: Supplementary file 2 [file Table_2.docx]

**Table S2**. Quality ratings for included studies

| Study (first author, year) | Item 1 | Item 2 | Item 3 | Item 4 | Item 5 | Item 6 | Item 7 | Item 8 | Item 9 | Item 10 | Item 11 | Item 12 | Item 13 | Item 14 | Total score for relevant questions |
| --- | --- | --- | --- | --- | --- | --- | --- | --- | --- | --- | --- | --- | --- | --- | --- |
| Braun et al. 2011 | **+** | **+** | - | + | - | + | + | + | + | - | + | N/A | - | - | 8.62 |
| Braun et al. 2012 | **+** | **+** | + | + | - | + | - | + | + | - | + | N/A | - | + | 9.69 |
| Cash et al.  2018 | **+** | **+** | + | + | + | + | + | + | + | - | + | N/A | - | + | 11.85 |
| Chandra et al.  2019 | **+** | **+** | - | + | - | + | + | + | + | - | + | N/A | - | - | 8.62 |
| Chang et al.  2014 | **+** | **+** | - | - | - | + | - | + | + | - | + | N/A | - | + | 7.54 |
| Collette et al.  2004 | **+** | **+** | + | - | - | + | + | + | + | - | + | N/A | - | + | 9.69 |
| Collins et al.  2017 | **+** | **+** | - | + | - | + | + | + | + | - | + | N/A | - | + | 9.69 |
| Geels et al.  2000 | **+** | **+** | + | + | - | + | + | + | + | + | + | N/A | - | - | 10.77 |
| Gottfried et al.  2020 | **+** | **+** | - | + | - | + | + | - | - | - | + | N/A | - | + | 7.54 |
| Innominato et al.  2009 | **+** | **+** | + | + | - | + | + | + | + | - | + | N/A | - | + | 10.77 |
| Innominato et al.  2012 | **+** | **+** | - | - | - | + | + | + | + | - | + | N/A | - | + | 8.62 |
| Innominato et al.  2015 | **+** | **+** | - | + | - | + | + | + | + | - | + | N/A | - | + | 9.69 |
| Kramer et al.  2000 | **+** | **+** | - | + | - | + | + | + | + | - | + | N/A | - | - | 8.62 |
| Kuo et al.  2020 | **+** | **+** | + | + | - | + | + | + | + | - | + | N/A | - | + | 10.77 |
| Levi et al.  2014 | **+** | **+** | - | + | - | + | + | + | + | - | + | N/A | - | + | 9.69 |
| Maisey et al.  2002 | **+** | **+** | - | - | - | + | + | + | + | - | + | N/A | - | + | 8.62 |
| Merli et al.  2004 | **+** | **+** | - | + | - | + | + | + | + | + | + | N/A | - | - | 9.69 |
| Mormont et al.  2000 | **+** | **+** | - | + | - | + | + | + | + | - | + | N/A | - | + | 9.69 |
| Naughton et al.  2002 | **+** | **+** | - | + | - | + | + | - | - | - | + | N/A | - | - | 6.46 |
| Nowak et al.  2004 | **+** | **+** | + | + | - | + | + | + | + | - | + | N/A | + | - | 10.77 |
| Palesh et al.  2014 | **+** | **+** | + | + | - | + | + | + | + | - | + | N/A | - | + | 10.77 |
| Robinson et al.  2012 | **+** | **+** | + | + | - | + | + | + | + | - | + | N/A | + | + | 11.85 |
| Roychowdhury et al.  2003 | **+** | **+** | - | + | - | + | + | + | + | - | + | N/A | - | - | 8.62 |
| Sullivan et al.  2006 | **+** | **+** | - | - | + | + | + | + | + | + | + | N/A | + | + | 11.85 |
| Teunissen et al.  2004 | **+** | **+** | - | + | - | + | + | + | + | + | + | N/A | - | - | 9.69 |
| Zhao et al.  2013 | **+** | **+** | + | + | - | + | + | + | + | + | + | N/A | - | - | 10.77 |

Notes: N/A indicates not applicable, and “+” indicates meeting criteria, whereas “– “ indicates not meeting criteria.

**NIH Quality Assessment Tool for Observational Cohort and Cross-Sectional Studies**

1. Was the research question or objective in this paper clearly stated?

2. Was the study population clearly specified and defined?

3. Was the participation rate of eligible persons at least 50%?

4. Were all the subjects selected or recruited from the same or similar populations (including the

same time period)? Were inclusion and exclusion criteria for being in the study prespecified and

applied uniformly to all participants?

5. Was a sample size justification, power description, or variance and effect estimates provided?

6. For the analyses in this paper, were the exposure(s) of interest measured prior to the

outcome(s) being measured?

7. Was the timeframe sufficient so that one could reasonably expect to see an association

between exposure and outcome if it existed?

8. For exposures that can vary in amount or level, did the study examine different levels of the

exposure as related to the outcome (e.g., categories of exposure, or exposure measured as

continuous variable)?

9. Were the exposure measures (independent variables) clearly defined, valid, reliable, and

implemented consistently across all study participants?

10. Was the exposure(s) assessed more than once over time?

11. Were the outcome measures (dependent variables) clearly defined, valid, reliable, and

implemented consistently across all study participants?

12. Were the outcome assessors blinded to the exposure status of participants?

13. Was loss to follow-up after baseline 20% or less?

14. Were key potential confounding variables measured and adjusted statistically for their impact

on the relationship between exposure(s) and outcome(s)?

Reference:

National Institutes of Health (NIH). Quality Assessment Tool for Observational Cohort and Cross-Sectional Studies. National Heart, Lung, and Blood Institute. https://www.nhlbi.nih.gov/health-topics/study-quality-assessment-tools. Accessed November 2, 2020.
